# Soporific Effect of Modified Suanzaoren Decoction and Its Effects on the Expression of CCK-8 and Orexin-A

**DOI:** 10.1155/2020/6984087

**Published:** 2020-06-16

**Authors:** Liang-Hui Zhan, Ying-Jie Dong, Ke Yang, Shan-Shan Lei, Bo Li, Xi Teng, Cong Zhou, Rong Luo, Qiao-Xian Yu, Hai-Ying Jin, Gui-Yuan Lv, Su-hong Chen

**Affiliations:** ^1^Collaborative Innovation Center of Yangtze River Delta Region Green Pharmaceuticals, No. 18, Chaowang Road, Xiacheng District, Zhejiang University of Technology, Hangzhou, Zhejiang 310014, China; ^2^Zhejiang Senyu Co., Ltd., No. 8 Wanmao Road, Choujiang Street, Yiwu, Zhejiang 322099, China; ^3^College of Pharmaceutical Science, No. 548, Binwen Road, Binjiang District, Zhejiang Chinese Medical University, Hangzhou, Zhejiang 310014, China

## Abstract

Suanzaoren decoction (SZRT), a classic Chinese herbal prescription, has been used as a treatment for insomnia for more than a thousand years. However, recent studies have found no significant effects of SZRT as a treatment for insomnia caused by gastric discomfort. Herein, we studied the effects of modified Suanzaoren decoction (MSZRD) on gastrointestinal disorder-related insomnia. The main constituents of MSZRD were spinosin (2.21 mg/g) and 6-feruloylspinosin (0.78 mg/g). A pentobarbital-induced animal model of insomnia showed that MSZRD shortened sleep latency and prolonged sleep time of the male Institute of Cancer Research (ICR) mice treated for 7 days with oral MSZRD. Sprague-Dawley male rats were treated daily with oral MSZRD or placebo for 11 days and then deprived of sleep for the last 4 days to establish a model of insomnia. Of note, MSZRD-treated animals had significantly improved body weight, organ index scores, and fecal moisture relative to placebo-treated animals, as well as reduced temperature. Sleep-deprived rats exhibited more exploratory behaviors in an open-field anxiety test; however, this effect was significantly reduced in MSZRD-treated animals. We found that MSZRD treatment decreased gastric acid pH, decreased the production of gastrin, pepsin, and Orexin-A, and increased the expression of MTL and CCK-8. Importantly, serum GABA concentration was increased by treatment with MSZRD, as reflected by a decreased Glu/GABA ratio. Treated animals had increased the expression of GAD1, GABARA1, and CCKBR but decreased the expression of Orexin R1. In summary, these results suggest that MSZRD has soporific and gastroprotective effects that may be mediated by differential expression of CCK-8 and Orexin-A.

## 1. Introduction

Insomnia is a common psychiatric disorder that can be caused by a wide range of factors such as medications, drug or alcohol abuse, medical conditions, depression, or anxiety [1]. Globally, it has been estimated that 10–30% of adults experience insomnia [2]. Two important monoamine neurotransmitters, *γ*-aminobutyric acid (*γ*-GABA) and Glutamate (Glu), have been increasingly recognized as playing an important role in the process of insomnia [3, 4]. Several prior experiments have shown that Glu, which is formed by PAG activity in GABAergic neurons, is converted by glutamate decarboxylase (GAD) to GABA [5, 6].

Insomnia may occur more frequently in patients with gastrointestinal symptoms. Up to 68% of patients in some clinical trials develop gastrointestinal complaints such as esophageal hyperalgesia and acid reflux, which may partially account for disordered sleeping [7, 8]. The stomach is particularly sensitive to stress, and insomnia can activate the sympathetic nervous system to cause the adrenal medulla to secrete catecholamine, which has a role in regulating the secretion of gastrin (GAS) and stomach acid [9]. Upon increased secretion of gastrin and stomach acid, the gastric mucosal barrier is weakened, potentially leading to tissue damage [10]. Interestingly, a large population-based study suggested a possible bidirectional association between sleep problems and gastrointestinal disease, wherein excessive secretion of gastric acid and pepsin may cause difficulty falling asleep and poor sleep quality [11, 12]. In turn, insomnia may lead to increased sympathetic activity that exacerbates gastrointestinal dysregulation. Although a number of pharmacologic and psychological treatments are available for insomnia, few are focused on the treatment of insomnia due to gastrointestinal discomforts. The use of benzodiazepines may even increase esophageal acid exposure time during sleep [13, 14]. Therefore, there is an urgent need for effective therapeutic options for the treatment of gastrointestinal disorder-related insomnia.

Cholecystokinin-8 (CCK-8) and Orexin-A are brain-gut peptides with various biological effects that are found not only in the gastrointestinal tract but also in the peripheral blood and hippocampus [15, 16]. CCK-8 has been shown to promote *γ*-GABA release from the synapses of the cerebral cortex and also to play a role in inhibiting gastric acid secretion [17, 18]. Similarly, Orexin-A is found in the gastrointestinal tract as well as the peripheral blood, and orexin has been reported to regulate food intake, wakefulness, and sleep-wake cycles [19]. Experimental research indicates that orexin facilitates the transformation of slow-wave sleep and rapid eye movement sleep (REMS) to the waking state by activating the orexin neurons, which are primarily located in the perifornical area and lateral hypothalamus [20]. Furthermore, inhibition of GABAergic inputs inhibits orexin neuron activity during NREM and REM sleep [21]. Orexin acts on the dorsal surface of the medulla through the vagus nerve to stimulate gastric acid secretion [22]. Therefore, it is possible that Orexin-A and CCK-8 form the biological basis between disturbed sleep and gastric discomfort.

Suanzaoren decoction (SZRT), a traditional Chinese herb remedy, includes Semen *Ziziphus jujuba* Mill. var. *spinosa* (Bunge) Hu ex H. F. Chou (Suanzaoren), *Poria cocos (*Schw.) Wolf (Fuling), *Ligusticum chuanxiong* Hort. (Chuanxiong), and *Glycyrrhiza uralensis* Fisch. (Gancao). SZRT has a long history of use as an effective treatment of insomnia [23]. Besides, SZRT could increase spontaneous sleep activity through mediating GABA (A) receptors [24]. However, we found that SZRT does not work well in ameliorating insomnia that is caused by gastric discomfort. Therefore, we created a modified SZRD (MSZRD) by adding *Dendrobium officinale* Kimura et Migo (Tiepishihu), which is believed to nourish the stomach and Yin and has been shown to protect against gastric mucosal injury in rats [25, 26]. We hypothesized that the combination of SZRD and Tiepishihu may provide synergistic effects for the treatment of insomnia due to gastric discomfort.

In this study, we first validated the soporific effect of MSZRD in a pentobarbital-induced sleep test. Then, we investigated its effects on the stomach by measuring the changes in the concentration of gastrointestinal hormones and assessing the histopathologic damage to the gastric mucosa. The soporific effects of MZSRD were further assessed biochemically by measuring the changes in the relative expression of monoamine neurotransmitters and behaviorally in an open field test. Last, we explored the potential roles of brain-gut peptides in insomnia due to gastric discomfort.

## 2. Materials and Methods

### 2.1. Chemicals and Reagents

*Ziziphus jujuba* Mill. var. *spinosa* (Bunge) Hu ex H. F. Chou (Suanzaoren) and *Dendrobium officinale* Kimura et Migo (Tiepishihu) were purchased from Zhejiang Senyu Co., Ltd (Zhejiang, China). Diazepam (G170301) was bought from Yunpeng Pharmaceutical Co., Ltd. (Shanxi, China). Suan Zao Ren Tang (0DM1058) was bought from Eu Yan Sang (Hongkong, China). Pentobarbital sodium (2018042001) was bought from Huaxia Chemical Reagent Co., Ltd. (Sichuan, China). GAS ELISA Kit, Orexin-A (OXA) ELISA Kit, and CCK-8 ELISA Kit (09/2019) were bought from Shanghai Enzyme-linked Biotechnology Co., Ltd. (Shanghai, China). Biochemical Assay Kit of Pepsin (20190910) was purchased from Nanjing Jiancheng Bioengineering Institute (Nanjing, China). Glu standards (20190113) and GABA (19042563) standards were purchased from Shanghai Sifeng Technology Co., Ltd. (Shanghai, China). Polyclonal antibodies of anti-CCKBR (BOS298BP87F) and anti-GABARA1 (11CM399) were purchased from Boster Biological Technology Co. Ltd. (California, USA). Orexin Receptor 1 Antibody (OXRA) (18370-1-AP) and GAD1 10408-1-AP) were bought from ProteinTech Group, Inc. (Wuhan, China). The standards of 6-feruloylspinosin (P17F8F29474) and spinosin (Y25O9H73433) were bought from Shanghai Yuanye Biotechnology Co., Ltd. (Shanghai, China).

### 2.2. MSZRD Component Analysis by HPLC

All separations were performed on the EC-C_18_ column (4.6 mm × 150 mm, 4 *μ*m) at 30°C on the Agilent 1260 HPLC system (Agilent Technologies, USA). The mobile phase was composed of solvent A (redistilled water) and solvent B (acetonitrile) with a linear gradient: 0–15 min (B 5–10%), 15–20 min (B: 10–15%), 20–40 min (B: 15–24%), 40–60 min (B:24–25%), 60–75 min (B: 25–26%), and 75–85 min (B: 26–5%). The DAD detection wavelength was set at 271 nm. Symmetrical peaks were obtained at a flow rate of 1.0 mL/min with a sample injection volume of 20 *μ*L [27].

### 2.3. Drug Preparation

MSZRD formula was prepared as 48 mL of decoction solution according to traditional methods, and ingredients were *Ziziphus jujuba* Mill. var. *spinosa* (Bunge) Hu ex H. F. Chou (Suanzaoren), *Poria cocos (*Schw.*) Wolf* (Fuling), *Ligusticum chuanxiong* Hort. (Chuanxiong), *Dendrobium officinale* Kimura et Migo (Tiepishihu), and *Glycyrrhiza uralensis* Fisch. (Gancao) (5 : 2 : 2 : 1 : 3), stored in the bridge of 4°C. Diazepam with dosages of 1.55 mg/kg and SZRT with 1.24 mg/kg were dissolved in distilled water.

### 2.4. Animals and Treatment

Male ICR mice (20 ± 2 g) and adult Sprague-Dawley rats (200 ± 10 g) were purchased from JOINN Laboratories (Suzhou) and raised in the animal room of Zhejiang University of Technology. They were given a free way to get food and water with humidity of 50 ± 10% at 23 ± 2°C or a 12 h light/dark cycle. All experiments were performed in accordance with the Regulations of Experimental Animal Administration issued by the Ministry of Science and Technology of the People's Republic of China. This experiment was approved by the ethics committee of Zhejiang University of Technology (SYXK (Zhe) 2017-0001).

In the pentobarbital-induced sleep test, 55 mg/kg pentobarbital (i.p.) was used as the hypnotic dose (sleep onset = 100%), and 35 mg/kg (i.p.) was used as the subhypnotic dose (sleep onset <10%). ICR mice were orally administered with MSZRD (14.4, 7.2, 3.6 g/kg) and diazepam (3 mg/kg), which were suspended in distilled water and administered 30 min before pentobarbital administration [28]. In the subhypnotic pentobarbital test, the percentage of mice that fell asleep was calculated as follows:

(1) $$\begin{array}{c} \text{sleep onset}\left(\%\right)=\frac{\text{no}.\text{ of mice falling asleep}}{\text{total no}.}\times 100\%. \end{array}$$

All Sprague-Dawley rats were divided into 7 groups randomly (*n* = 7–9): (1) control group, (2) model group, (3) MSZRD-H group (7.44 g/kg, MSZRD of high dose), (4) MSZRD-M group (3.72 g/kg, MSZRD of medium dose), (5) MSZRD-L group (1.86 g/kg, MSZRD of low dose), (6) DZP group (1.55 mg/kg, diazepam), and (7) SZRT (1.24 g/kg, Suan Zao Ren Tang). The rats from the control group and model group were given distilled water 1 mL/100 g weight and the rest were given the same volume/weight medicine, respectively. The method of drug administration was adopted while building the model in this experiment. And all of the groups except the control group were sleep deprived for 4 days [29] and given hot drugs which consisted of *T. hypoglaucum* and cinnamon by gavage for 7 days [30] to build gastrointestinal disorder-related insomnia model. Then, gastric tissues and brain tissue were collected followed by washing with the cold saline, and blood plasma was collected by the abdominal aortic method after all rats were sacrificed, stored in −80°C bridge.

Sleep deprivation was induced by placing rats in modified water-filled multiple platforms (125 cm × 44 cm) that consisted of 8 small circular platforms (6.5 cm, in diameter) which were submerged to about 1–2 cm above the water surface. The platforms were located at a distance, so rats could move freely from one to another and balance on the platforms. Due to natural REM's muscle paralysis, the rats would come into contact with the water and awaken. The rats had free access to clean water and food pellet baskets hanging from the aquarium cover. For the control group, using larger platforms (14 cm in diameter) caused to fall sleep without falling down. The SD period lasted for 96 hours [31].

### 2.5. Measurement of Weight, Temperature, and Thermal Imaging

The weights of the rats were measured every three days. Using an Anal thermometer to detect the anal temperature during the experiment. Thermal imaging was shot by thermal imaging camera and the neck temperature was analyzed by Imagine Pro.

### 2.6. Open-Field Test

After the final drug administration for 30 min, rats of each group were subjected to an open-field test. The rats were placed in the laboratory for 10 min to adapt to the environment before being placed into a wooden open field box. Synchronized video and timing recordings were collected to observe rat activity within the first 5 minutes [32]. Autonomous activity test index: total movement distance (unit: cm), maximum velocity (unit: cm/s), and track chart.

### 2.7. Biochemical Assays

The activity of GAS, pepsin, MTL, OXA, and CCK-8 was detected by enzyme-linked immunosorbent assay (ELISA) and Biochemical tests according to the protocol provided by the manufacturer. The pH of stomach acid was measured by pH meter (Mettler Toledo, Zurich, Switzerland).

### 2.8. HPLC Analysis of *γ*-GABA and Glu

First, 0.5 mL perchloric acid (0.4 mol/L) was added to serum to remove protein, and then centrifugation was done to obtain a supernatant. Following that, the supernatant was added to 0.5 mL Na_2_CO_3_ (1 mol/L) to get the sample for HPLC detection.

Amino acids were assayed by HPLC after precolumn derivatization in a timed reaction with O-phthalaldehyde plus mercaptoethanol [33]. Derivatized samples (100 *μ*L) were injected into EC-C18 column (4.6 mm × 150 mm, 4 *μ*m) at 30°C with a mobile phase of acetonitrile-water (50 : 50, A) and sodium acetate (0.05 mol/L, B). The DAD detection wavelength was set at 338 nm. Symmetrical peaks were obtained at a flow rate of 1.0 mL/min.

### 2.9. Histological Evaluations and Immunohistochemistry

The brain tissue and gastric tissue were fixed in 4% paraformaldehyde solution for 24 h, dehydrated with 95% ethanol, and embedded in paraffin. Embedded sections were cut using the microtome at a thickness of 4 *μ*m. And paraffin-embedded tissue sections were stained with hematoxylin and eosin (H&E) [34] to evaluate the brain and gastric mucosa damage. Nissl staining was used to analyze Nissl bodies in nerve cells, and the paraffin-free tissues were stained with 0.5% toluidine blue water solution at 56 degrees Celsius for 10 minutes, and the deparaffinized tissue was stained with the method of immunohistochemical staining. They were incubated with anti-GABAAR, GAD1, CCKBR, and OXR1 primary antibodies. A secondary antibody HRP conjugated goat anti-rabbit IgG was added. The signals were visualized by DAB staining and the nuclei were counterstained with hematoxylin. Positive staining presented yellow color under a microscope. All staining was photographed with the biological microscope, 200x augmentation for ten fields [35].

### 2.10. Western Blot Assay

Western blot was used to detect GABAAR, GAD1, CCKBR, and OXR1. The proteins were extracted with radioimmunoprecipitation (RIPA) buffer, and the concentration was quantitated by BCA assay. The total protein (100 *μ*g) was separated on 10% resolving SDS-PAGE gel and 5% stacking gel and transferred to PVDF membrane. Next, membranes were blocked with 5% skim milk in Tris-buffered saline containing 0.1% Tween-20 (TBST) for 2 h, incubated with the primary antibody overnight at 4°C, and then incubated with horseradish peroxidase-conjugated secondary antibody after washing with TBST. Membranes were visualized using an enhanced chemiluminescence (ECL) detection system. The density of each band was estimated using the Image Lab software. All target proteins were normalized against the loading control GAPDH.

### 2.11. Statistical Analysis

Each experiment was performed at least three times, and all results were presented as means ± SD. Results were analyzed with IBM SPSS Statistics 19.0 (SPSS Inc., NY, USA). Significant differences were determined by a Student's *t*-test and one-way analysis of variance (ANOVA) (^*∗*^*P* < 0.05 or ^*∗∗*^*P* < 0.01). *χ*^2^ test was used for the subhypnotic dose of pentobarbital test and gastric mucosal injury rate. Differences were considered statistically significant at ^*∗*^*P* < 0.05.

## 3. Results

### 3.1. HPLC Result of MSZRD Detection

**Figure 1:**
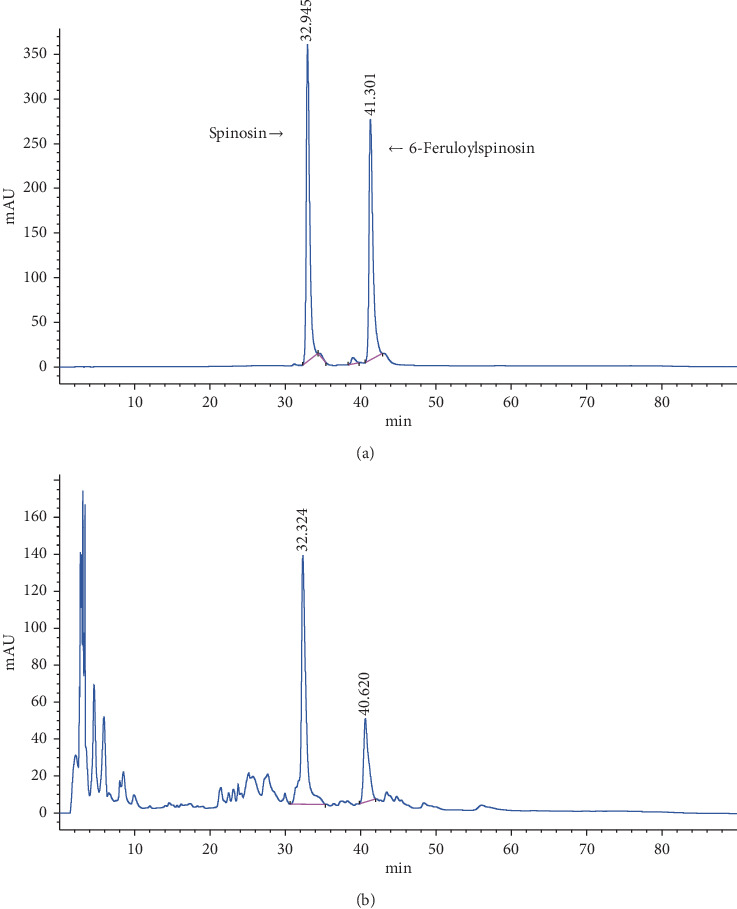
High-performance liquid chromatogram of the standard of spinosin and 6-feruloylspinosin and MSZRD at 271 nm. Standard of spinosin and 6-feruloylspinosin (a) and MSZRD (b) were measured for three times by HPLC.

HPLC analysis was used to identify the MSZRD (Figures 1(a) and 1(b)). According to the methods described above, standard curves of spinosin and 6-feruloylspinosin were obtained which were *y* = 27.108*x* − 80.704 (*R*^2^ = 0.997) and *y* = 22.723*x* + 285.46 (*R*^2^ = 0.9981). And the sample of MSZRT produces two prominent peaks corresponding to spinosin (2.21 ± 0.001 mg/g) and 6-feruloylspinosin (0.78 ± 0.007 mg/g).

### 3.2. Onset and Duration of Sleep in the Pentobarbital-Induced Sleep Test

**Figure 2:**
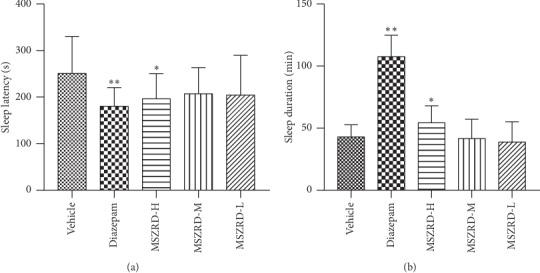
The sleep latency and sleep duration to pentobarbital-induced sleep in mice. Sleep latency (a) and sleep duration (b) were measured. Data are expressed as the mean ± SD of three independent experiments. ^*∗*^*P* < 0.05 and ^*∗∗*^*P* < 0.01 compared with the vehicle group.

In order to investigate the hypnotic effects of MSZRD, sleep onset and duration of pentobarbital-induced sleep in mice were measured. As is shown in Table 1, in mice treated with subhypnotic doses of pentobarbital (35 mg/kg), MSZRD showed a tendency to improve the sleep onset in mice, but it was not statistically significant. In mice with a hypnotic dose of pentobarbital (55 mg/kg) treatment, MSZRD at 14.4 g/kg could obviously reduce the sleep latency (*P* < 0.05) and improve the sleep duration of mice (*P* < 0.05) (Figures 2(a) and 2(b)). The results showed that MSZRD had hypnosis effects.

### 3.3. Changes in Weight and Pyresis following the Treatment with MSZRD

**Figure 3:**
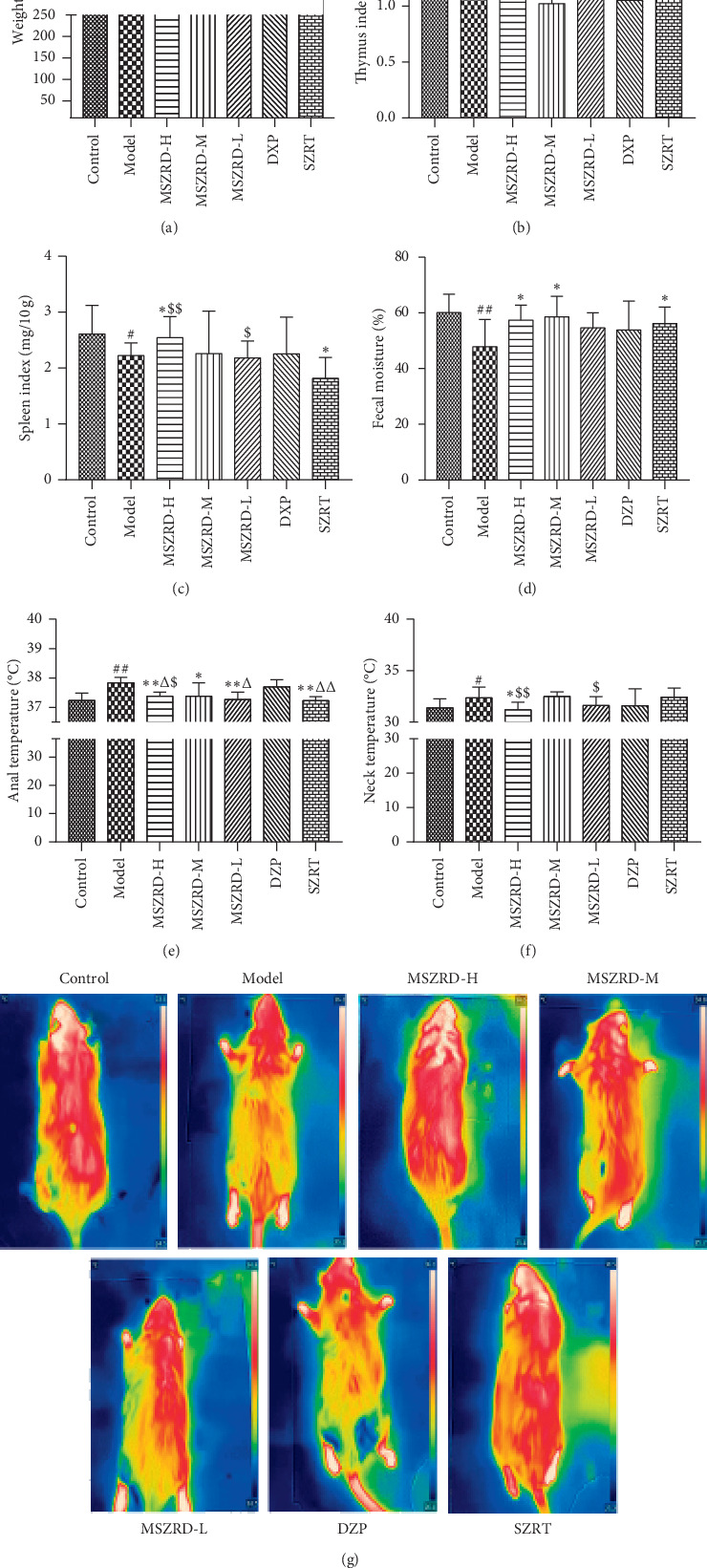
The comparison of weight as well as organ index in each group and the antipyretic effects of MSZRD. The weight (a), thymus (b), and spleen (c) mass were gained before the rats were sacrificed. Fecal moisture (d) and anal temperature (e) were measured on day 7 of the experimental process. Thermal imaging was photographed (g) by thermal imagine camera and statistical analysis on the neck temperature (f) by Imagine Pro. Data are expressed as the mean ± SD of three independent experiments. ^#^*P* < 0.05 and ^##^*P* < 0.01 compared to the control group; ^*∗*^*P* < 0.05 and ^*∗∗*^*P* < 0.01 compared to the model group; ^Δ^*P* < 0.05 and ^ΔΔ^*P* < 0.01 compared to the DZP group; ^$^*P* < 0.05 and ^$$^*P* < 0.01 compared to the SZRT group.

Body weights were measured daily and organ weights were measured at the end of the experiment to investigate the effects of MSZRT on immunity. Compared with the healthy control group, the weight, thymus index, and spleen index of the untreated model group were significantly decreased (*P* < 0.05, 0.01). However, as expected, treatment with a high dose of MSZRD (MSZRD-H) protected against this decline in body weight and organ indices (*P* < 0.05). Compared with the DZP group, the MSZRD-H also significantly improved the thymus index (*P* < 0.01). Furthermore, the thymus and spleen indices were both significantly increased after treatment with MSZRD compared to the SZRT group (*P* < 0.05, 0.01) (Figures 3(a)–3(c)).

To investigate the antipyretic effects of MSZRD, we measured the temperature of the anus and neck every day at approximately 10 am. Animals treated with MSZRD had significantly lower temperatures than model animals, similar to the control group (*P* < 0.05). Fecal moisture content is a validated indicator of antipyretic effects in rat models of MSZRD. We measured fecal moisture in samples collected immediately after excretion and found that fecal moisture content was significantly reduced relative to the untreated model group (*P* < 0.01). Compared with the DZP group, MSZRD-treated animals had significantly lower average anal temperatures (*P* < 0.05). Similarly, the average neck temperature in MSZRD-treated animals was also lower than that in the SZRT group (*P* < 0.05, 0.01).

### 3.4. Open-Field Test Results

**Figure 4:**
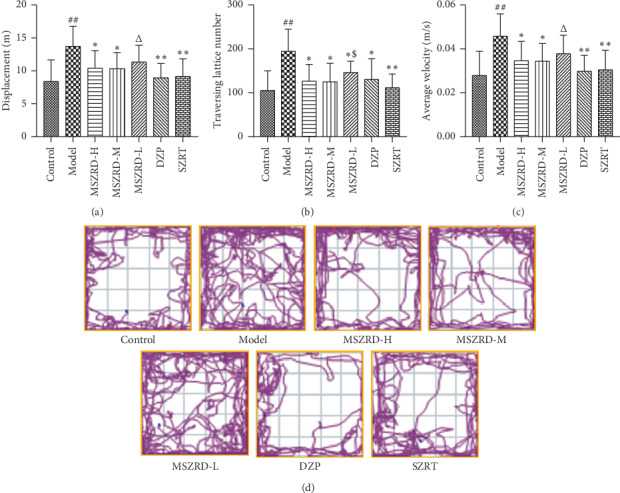
Assessment of the anxious behavior following MSZRD treatment during sleep deprivation (SD). Displacement (a), traversing lattice number (b), average velocity (c), and tract plot (d) were collected during the open field test. Data are expressed as the mean ± SD of three independent experiments. ^#^*P* < 0.05 and ^##^*P* < 0.01 compared to the control group; ^*∗*^*P* < 0.05 and ^*∗∗*^*P* < 0.01 compared to the model group; ^Δ^*P* < 0.05 and ^ΔΔ^*P* < 0.01 compared to the DZP group; ^$^*P* < 0.05^$$^*P* < 0.05 and ^$$^*P* < 0.01 compared to the SZRT group.

An open-field test was used to assess the anxious behavior of sleep deprivation rats. The results revealed that model group rats had an improvement in displacement, average velocity, and traversing lattice number which is remarkable and also showed the disordered track, compared to the control group (*P* < 0.01), while displacement, traversing lattice number, and average velocity were obviously reduced in Sprague-Dawley rats treated with MSZRD-H and MSZRD-M compared with the model group (*P* < 0.05) (Figures 4(a)–4(d)). The open-field test indicated that MSZRD could help relieve the anxiety of insomnia rats.

### 3.5. Analysis of Brain Neurotransmitters

**Figure 5:**
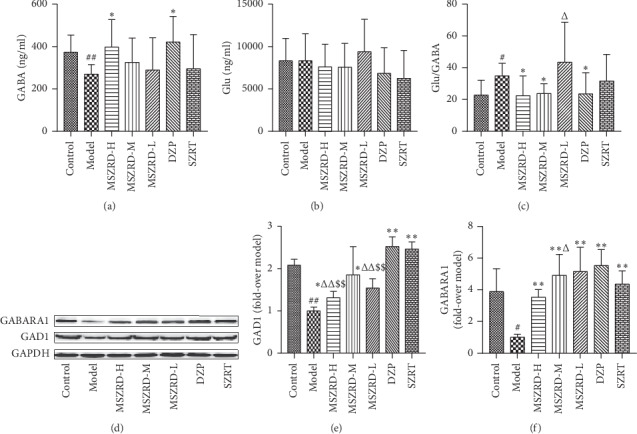
Effects of MSZRD on the level of *γ*-GABA as well as Glu and the representative western blot images of GABARA1 and GAD1. The levels of *γ*-GABA (a) and Glu (b) were detected by HPLC, and their ratio was calculated (c). The expression of GABARA1 and GAD1 was measured by western blot (d). Statistical analysis of GAD1 and GABARA1 (e-f). Data are expressed as the mean ± SD of three independent experiments. ^#^*P* < 0.05 and ^##^*P* < 0.01 compared to the control group; ^*∗*^*P* < 0.05 and ^*∗∗*^*P* < 0.01 compared to the model group; ^Δ^*P* < 0.05 and ^ΔΔ^*P* < 0.01 compared to the DZP group; ^$^*P* < 0.05^$^*P* < 0.05 and ^$$^*P* < 0.01 compared to the SZRT group.

The concentrations of GABA and Glu in serum were detected by HPLC. And the results showed that the content of GABA was dramatically reduced in the model group compared with the control group (*P* < 0.01), along with the higher Glu/GABA ratio (*P* < 0.05), but the Glu has no statistical differences. As expected, MSZRD and diazepam treatment successfully increased the production of GABA and reduced the Glu/GABA ratio compared to the model group (*P* < 0.05). But the Glu/GABA ratio in the MSZRD-L group was higher than that in the DZP group (*P* < 0.05) (Figures 5(a)–5(c)). Correspondingly, the expression of GABARA1 and GAD1 in the hippocampus in the model group was lower than that in the control group (*P* < 0.05, 0.01). However, MSZRD and diazepam treatment increased the expression level of GAD1 and GABARA1 compared with the model group, significantly (*P* < 0.05, 0.01). But the production of GAD1 and GABARA1 in the MSZRD group was lower than that in the DZP group as well as the SZRT group (*P* < 0.05, 0.01) (Figures 5(d)–5(f)).

### 3.6. Histological Evaluation of Brain Tissue

**Figure 6:**
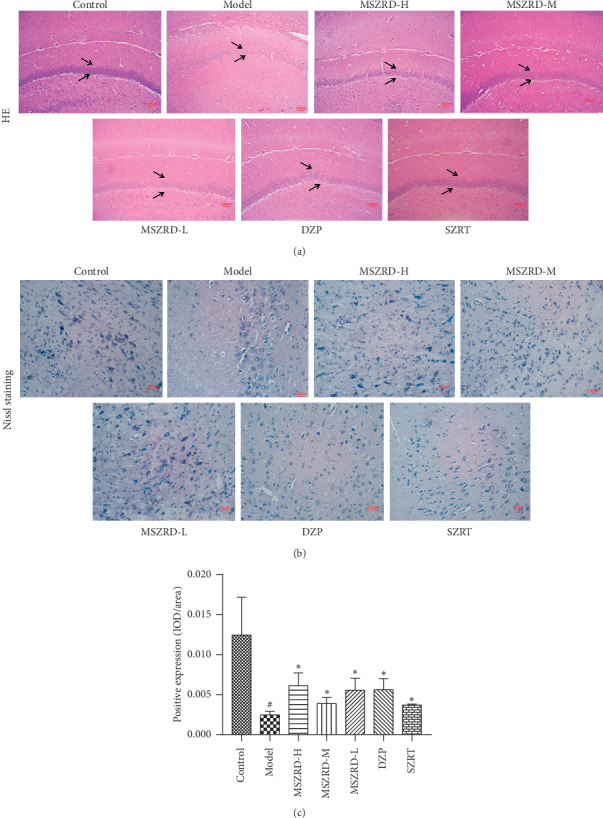
Effects of MSZRD on histological evaluation of brain tissue with H&E staining and Nissl staining. H&E staining, “⟶” points to hippocampal (a), magnification (100x); Nissl staining, “⟶” points to Nissl body (b) which was quantified as IOD/area by Imagine Pro (c), magnification (400x). Data are expressed as the mean ± SD of three independent experiments. ^#^*P* < 0.05 and ^##^*P* < 0.01 compared to the control group; ^*∗*^*P* < 0.05 and ^*∗∗*^*P* < 0.01 compared to the model group.

As is shown, nerve cells were arranged in a well-organized way in the hippocampus and a number of nerve cells were with large and round nuclei in the control group. But the arrangement of hippocampal neurons was disordered and loose, and some neurons decreased and the normal morphology disappeared, as well as Nissl body was lost in the model group. In addition, the number of surviving neurons stained by Nissl staining presented a significantly decreased integrated optical density (IOD) in the model group contrary to the control group (*P* < 0.05). Compared to the model group, MSZRD and diazepam improved the pathological changes with a regular arrangement of hippocampal neurons and numerous Nissl body (*P* < 0.05) (Figures 6(a)–6(c)).

### 3.7. Gastrointestinal Effects of MSZRD on Sleep-Deprived Rats

**Figure 7:**
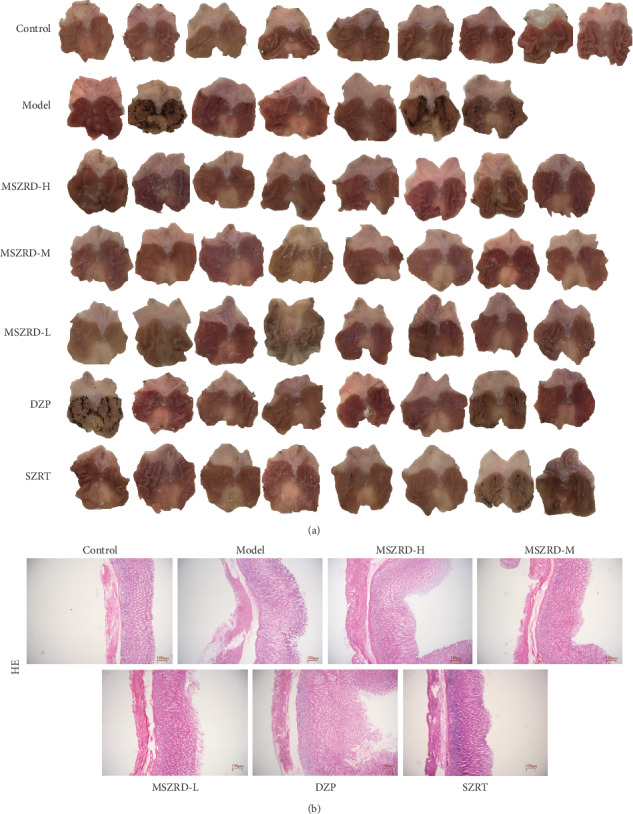
Effects of MSZRD on macroscopic images of the gastric mucosal and histological evaluation. The images of gastric mucosal tissues (a). H&E staining (b) (100x). ○ stands for dead rats which were sacrificed during the experiment.

Sleep deprivation as well as hot drugs produced gastric mucosal injury of about 42.86% in the model group compared to the control group without gastric lesions (*P* < 0.05). But, treatment with a medium-high dose of MSZRD dismissed the lesion totally, contrary to the model group (*P* < 0.05). And rats administrated with DZP and SZRT still suffered the gastric damage rate of 25.00% and 12.50% (Table 2). H&E staining of gastric tissue showed that the gastric mucosa of rats in the normal control was smooth and the arrangement of basal epithelial cells was tight. However, the microscopic appearance of the lesions in the gastric mucosa in the model and DZP groups has epithelial cell separation and heeding. As expected, the pathological injury of gastric mucosa had amelioration with MSZRD treatment, obviously (Figure 7).

### 3.8. Effects of MSZRD on Gastric Acid Secretion and Gut Hormones

**Figure 8:**
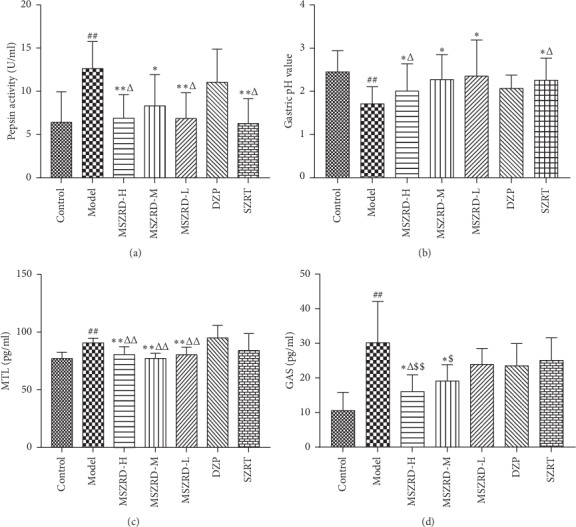
Effects of MSZRD on gastric acid secretion and gut hormones. Pepsin activity (a), MTL (c), and GAS (d) levels were measured by Biochemical Kit and ELISA kits. Gastric pH value (b) was detected by using a pH meter. Data are expressed as the mean ± SD of three independent experiments. ^#^*P* < 0.05 and ^##^*P* < 0.01 compared to the control group; ^*∗*^*P* < 0.05 and ^*∗∗*^*P* < 0.01 compared to the model group; ^Δ^*P* < 0.05 and ^ΔΔ^*P* < 0.01 compared to the DZP group; ^$^*P* < 0.05 and ^$$^*P* < 0.01 compared to the SZRT group.

It was found that the levels of pepsin and GAS were improved rapidly in the model group, with lower MTL and gastric acid PH value, compared with the control group (*P* < 0.01), while MSZRD with medium to high dose treatment could reduce the activity of pepsin, GAS, and MTL and alleviate the change of gastric acid PH value (*P* < 0.05, 0.01). Do note that pepsin activity and the level of GAS as well as MTL in MSZRD were lower than those in the DZP group (*P* < 0.05). Also, the production of GAS was remarkably decreased in MSZRD than in the SZRT group. Correspondingly, gastric PH value in MSZRD-H was less than that in the DZP group (*P* < 0.05) (Figures 8(a)–8(d)). The results indicated that MSZRD played an important role in gastroprotective effects by inhibiting the aggressive factor.

### 3.9. MSZRD Affects Orexin-A and CCK-8 Expression

**Figure 9:**
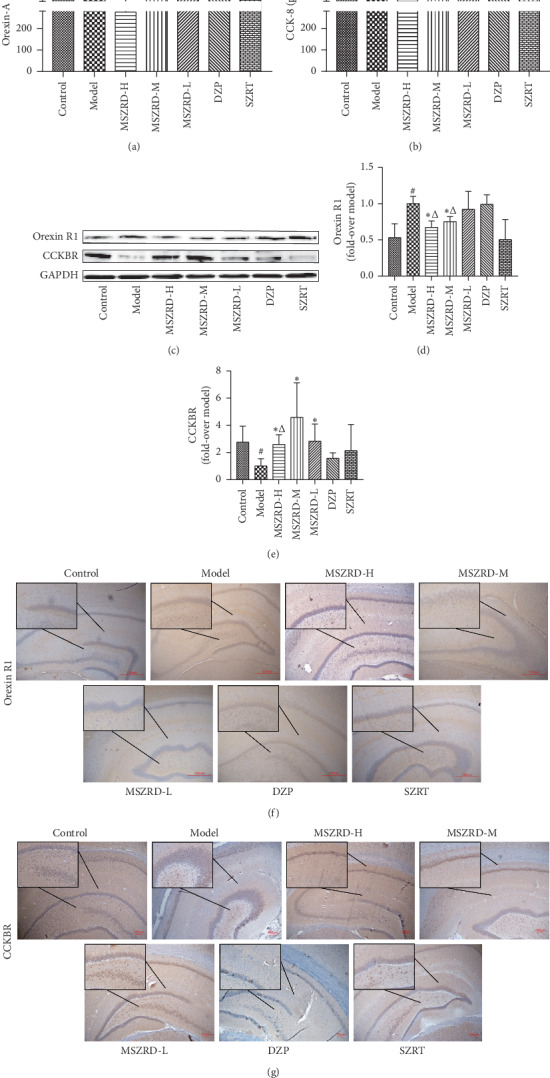
Effects of MSZRD on the level of CCK-8 and Orexin-A and the expression of orexinR1 and CCKBR. The level of Orexin-A (a) and CCK-8 (b) in serum was detected by ELISA kits. The production of orexinR1 and CCKBR (c) was measured by western blot. Statistical analysis on orexinR1 and CCKBR (d, e). The expression of orexinR1 (f) and CCKBR (g) in brain tissues was examined by immunohistochemical analysis (200x, brown-yellow granules indicate positive reaction). Data are expressed as the mean ± SD of three independent experiments. ^#^*P* < 0.05 and ^##^*P* < 0.01 compared to the control group; ^*∗*^*P* < 0.05 and ^*∗∗*^*P* < 0.01 compared to the model group; ^Δ^*P* < 0.05 and ^ΔΔ^*P* < 0.01 compared to the DZP group; ^$^*P* < 0.05 and ^$$^*P* < 0.01 compared to the SZRT group.

To investigate the effects of MSZRD on the brain-gut peptide, the content of Orexin-A as well as CCK-8 in serum and the expression of OrexinR1 with CCKBR in hippocampus were detected. The results showed that the production of Orexin-A and OrexinR1 was increased in the model group in comparison to the control group (*P* < 0.05). By contrast, MSZRD treatment could decrease the expression of Orexin-A and OrexinR1 (*P* < 0.05). What's more, the expression of Orexin-A and OrexinR1 in MSZRD was obviously lower than that in the DZP group (*P* < 0.05, 0.01) and the Orexin-A content, even less than that in the SZRT group (*P* < 0.01) (Figures 9(a), 9(c), 9(d), and 9(f)). Similarly, CCK-8 and CCKBR were decreased in the model group in contrast to the control group (*P* < 0.05, 0.01). However, compared with the model group, MSZRD treatment could improve the expression of CCK-8 and CCKBR (*P* < 0.05, 0.01). And the level of CCK-8 and CCKBR in the MSZRD group was even higher than that in the DZP group (*P* < 0.05, 0.01) (Figures 9(b), 9(c), 9(e), and 9(g)).

## 4. Discussion

In this study, we first created a modified Suanzaoren decoction (MSZRD) and validated its soporific and gastroprotective effects. Then, we explored the effects of brain-gut peptides in a rat model of insomnia with gastrointestinal involvement.

Insomnia is a common complaint that can be closely bidirectionally related to gastrointestinal symptoms [36]. Thus, it is ideal to simultaneously improve gastrointestinal function and to restore the natural sleep-wake cycle for individuals with insomnia due to gastrointestinal complaints [37]. In the Traditional Chinese Medicine (TCM) subtype medium of insomnia with stomach discomforts, stomach heat syndrome is characterized by constipation, elevated temperature, and a constellation of related symptoms [38]. Herein, we found that MSZRD has the function of clearing stomach and purging heat resulting from serving hot drugs [30]. However, treatment with DZP was less able to attenuate fever. Clinically, insomnia typically results in decreased immune function, manifesting in the form of decreased weight and immune organ mass [39]. However, we found that DZP, despite being a fundamental element in the therapeutic approach to managing insomnia, did not significantly protect against decreases in bodyweight or immune organ weight [40]. In contrast, treatment with MSZRD did improve bodyweight as well as the spleen and thymus indices relative to untreated model animals.

Anxiety is highly interconnected with insomnia because sleep deprivation lowers the psychological threshold for stress tolerance [41, 42]. In this work, we assessed the anxiety of Sprague-Dawley rats in an open field test and found that MSZRD significantly reduced behavioral signs of anxiety relative to untreated model animals. A significant body of evidence has suggested that insomnia and sleep deprivation may enhance gastric mucosal injury and the development of ulcers [43]. In this study, a rat insomnia model was built via sleep deprivation for 96 h, which resulted in clear macroscopic damage as well as the microscopic development of lesions in the gastric mucosa. Further, it has previously been shown that insomnia can induce hippocampal injury and Nissl substance loss. This prior finding is in good agreement with our results, because it has been shown that sleep deprivation causes hippocampus damage by increasing oxidative stress, eventually leading to neuronal apoptosis and Nissl substance loss in the brain [44, 45]. However, we found that treatment with MSZRD attenuated damage to the gastric mucosal and hippocampus.

Monoamine neurotransmitters play an important role in the sleep-wake cycle [46]. GABA, which is the main inhibitory neurotransmitter in the CNS, tends to increase sleep time [28]. In addition, some reports have found that the level of GABA is decreased significantly after sleep deprivation [47, 48]. Furthermore, GAD (glutamate decarboxylase), as one of the major enzymes converting Glu into GABA, is also crucial for the sleep-wake cycle [49]. Thus, we measured the concentrations of GABA and Glu in the serum as well as the expression of GAD in the hippocampus. Our results showed that sleep deprivation for 96 h decreased the expression of both GABA and GAD and increased the Glu/GABA ratio; however, the treatment with MSZRD reversed these changes. Of note, GABA acts through GABA receptors, of which GABAR1 is the most important receptor in the context of sleep [50]. When GABA or another agonist binds to the GABAA receptor, it triggers the influx of chloride ions into neuronal cells, which reduces activity. Therefore, the activation of GABAA receptors is beneficial for sleep [51]. Our results showed that MSZRD upregulated the expression of GABAR1. This indicates that GABA might be of primary importance in the mechanism of the soporific effects of MSZRD.

Insomnia and sleep deprivation can cause increased production of gastrin, regulating gastric acid secretion and motilin (MTL) that activate pepsin. [52, 53]. If gastric acid levels are too high, the gastric mucosal barrier can become compromised, leading to potential tissue damage [43]. Interestingly, Dimarino et al. found that inhibiting acid secretion reduces arousal during sleep and prolongs total sleep time [12]. Since benzodiazepines decrease basal esophageal sphincter (LES) pressure in humans, this class of drugs might worsen gastric discomforts and this study verified that effect. We also found that MAZRD inhibited the production of GAS, pepsin, MTL, and gastric acid, restoring healthy gastrointestinal function. These results suggested that MSZRD may be a promising therapeutic candidate for insomnia due to gastric discomfort due to its ability to both improve sleep quality and restore gastric function.

Prior studies have shown that one of the insomnia core resulted from an imbalance in the brain-gut [54]. Therefore, we conducted a preliminary study of the brain-gut peptides CCK-8 and Orexin-A. Lanza M. et al. reported that CCK-8, the most highly expressed CCK in the human body that is mainly present in the central nervous system, can not only facilitate the release of *γ*-aminobutyric acid (GABA) in the brain, act as an inhibitory neurotransmitter, and prolong the NREMS via CCKB receptors [55] but also play a physiological role in inhibiting gastric acid secretion [17]. Experimental evidence has indicated that MSZRD increases the expression of CCK-8 and CCKBR, but diazepam has no effect. Importantly, we found that MSZRD decreased gastric acid secretion and the activity of pepsin. Orexin-A has also been shown to be involved in the central control of gastric acid and pepsin secretion [56]. A large prior study showed that Orexin-A signaling is important for sleep behavior, especially for staying awake [57]. Interestingly, MSZRD increased the production of Orexin-A and downregulated the expression of the Orexin-A receptor in good agreement with other research results. However, diazepam and SZRT did not appear to inhibit Orexin-A, potentially because of the increased secretion of GAS in these two groups.

In summary, treatment with MSZRD provided the benefits of sleep improvement and protection against gastrointestinal disorders in a rat model of insomnia, possibly through a mechanism related to Orexin-A and CCK-8. We hypothesize that MSZRD improved insomnia by inhibiting acid secretion to relieve gastric discomforts and thus improved sleep quality. However, the specific mechanisms involved in this pathway remain a topic for future studies, especially the question of whether MSZRD acts by inhibiting CCK-8 and Orexin-A.

## 5. Conclusion

**Figure 10:**
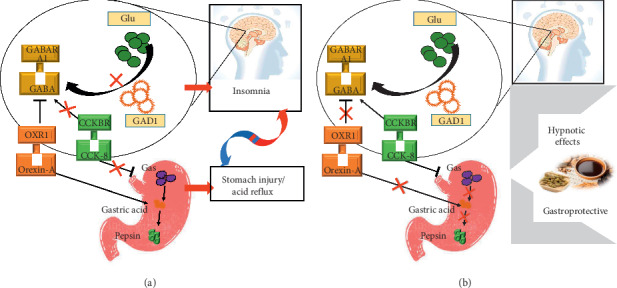
Soporific effect of modified Suanzaoren decoction and its effects on the expression of CCK-8 and Orexin-A. (a) Before treatment. (b) After treatment.

We found that MSZRD had a soporific effect and gastroprotective effect in a rat model of gastrointestinal discomfort-related insomnia. These preliminary findings suggest that MSZRD might regulate the brain-gut peptides (CCK-8↑, Orexin-A↓) to increase the content of GABA and the expression of GABARA1 and GAD1 in order to improve insomnia, which is manifested as decreased latency of sleeping time and prolonged duration of sleeping time alongside a reduction in anxious behavior. Additionally, MSZRD may affect the brain-gut peptides (CCK-8↑, Orexin-A↓) to reduce the production of GAS, MTL, and the secretion of gastric acid and pepsin, leading to a generally gastroprotective effect. In contrast, diazepam aggravated gastric acid secretion. These results suggest that MSZRD may be a promising natural agent to serve as the basis for the development of products for the therapeutic management of insomnia related to gastrointestinal discomfort (Figure 10).

## Figures and Tables

**Table 1:** Effects of MSZRD on sleep onset induced by subhypnotic dosage of pentobarbital.

Group	Dosage (g/kg, ig)	No. of mice falling asleep/total	Sleep onset (%)
Vehicle	—	1/10	10
Diazepam	0.003	9/10	90^*∗∗*^
	14.4	4/10	40
MSZRD	7.2	3/10	30
	3.6	4/10	40

Thirty minutes after the administration of distilled water, diazepam, and MSZRD, pentobarbital (35 mg/kg, i.p.) was given to mice. (*χ*^2^ test). Compared with the vehicle group, ^*∗*^*P* < 0.05 and ^*∗∗*^*P* < 0.01.

**Table 2:** Gastric mucosa injury rate of sleep deprivation rats.

Group	Dosage (g/kg, ig)	No. of injuries/total	Gastric mucosa injury rate (%)
Control	—	0/9	0.00
Model	—	3/7	42.86^#^
MSZRD-H	7.44	0/8	0.00^*∗*^
MSZRD-M	3.72	0/8	0.00^*∗*^
MSZRD-L	1.86	1/8	12.50
DZP	1.55 × 10^−3^	2/8	25.00
SZRT	1.24	1/8	12.50

The number of gastric mucosal injuries of sleep deprivation rats at the end of the experiment. ^#^*P* < 0.05 and^##^*P* < 0.01 compared to the control group; ^*∗*^*P* < 0.05 and ^*∗∗*^*P* < 0.01 compared to the model group (*χ*^2^ test).

## Data Availability

The data used to support the findings of this study are included within the article.

## Supplementary Materials

Supplementary Materials This experiment is mainly divided into the following three parts: first, using HPLC to detect the main ingredients in modified Suanzaoren decoction; second, evaluating its hypnotic effect through the pentobarbital-induced animal model of insomnia; and finally, establishing insomnia related to gastrointestinal diseases through sleep deprivation for 4 days and administration of hot drugs for 7 days, to evaluate the hypnotic effect of the modified Suanzaoren decoction and its possible mechanism.

## References

[1] Oh D.-R., Kim Y., Jo A. (2019). Sedative and hypnotic effects of Vaccinium bracteatum thunb. through the regulation of serotonegic and GABAA-ergic systems: involvement of 5-HT1A receptor agonistic activity. *Biomedicine & Pharmacotherapy*.

[2] Bhaskar S., Hemavathy D., Prasad S. (2016). Prevalence of chronic insomnia in adult patients and its correlation with medical comorbidities. *Journal of Family Medicine and Primary Care*.

[3] Takeshima K., Yamatsu A., Yamashita Y. (2014). Subchronic toxicity evaluation of *γ*-aminobutyric acid (GABA) in rats. *Food and Chemical Toxicology*.

[4] Zhang R., Yang Y. S., Liu X. C. (2016). Correlation study of basic Chinese medicine syndromes and neurotransmitter levels in patients with primary insomnia. *Chinese Journal of Integrative Medicine*.

[5] Waagepetersen H. S., Sonnewald U., Schousboe A. (2003). Compartmentation of glutamine, glutamate, and GABA metabolism in neurons and astrocytes: functional implications. *The Neuroscientist*.

[6] Bak L. K., Schousboe A., Waagepetersen H. S. (2006). The glutamate/GABA-glutamine cycle: aspects of transport, neurotransmitter homeostasis and ammonia transfer. *Journal of Neurochemistry*.

[7] Fass R., Fullerton S., Tung S., Mayer E. A. (2000). Sleep disturbances in clinic patients with functional bowel disorders. *The American Journal of Gastroenterology*.

[8] Fujiwara Y., Arakawa T., Fass R. (2012). Gastroesophageal reflux disease and sleep disturbances. *Journal of Gastroenterology*.

[9] Caldara R., Barbieri C., Piepoli V., Borzio M., Masci E. (1985). Effect of L-dopa with and without inhibition of extra cerebral dopa decarboxylase on gastric acid secretion and gastrin release in man. *Gut*.

[10] Engevik A. C., Kaji I., Goldenring J. R. (2020). The physiology of the gastric parietal cell. *Physiological Reviews*.

[11] Jansson C., Nordenstedt H., Wallander M. A. (2009). A population-based study showing an association between gastroesophageal reflux disease and sleep problems. *Clinical Gastroenterology and Hepatology*.

[12] Johnson D. A., Orr W. C., Crawley J. A. (2005). Effect of esomeprazole on nighttime heartburn and sleep quality in patients with GERD: a randomized, placebo-controlled trial. *The American Journal of Gastroenterology*.

[13] Schneider J., Patterson M., Jimenez X. F. (2019). Beyond depression: other uses for tricyclic antidepressants. *Cleveland Clinic Journal of Medicine*.

[14] Gagliardi G. S., Shah A. P., Goldstein M. (2009). Effect of zolpidem on the sleep arousal response to nocturnal esophageal acid exposure. *Clinical Gastroenterology and Hepatology*.

[15] Brzozowski T., Konturek P. C., Sliwowski Z. (2008). Gastroprotective action of orexin-A against stress-induced gastric damage is mediated by endogenous prostaglandins, sensory afferent neuropeptides and nitric oxide. *Regulatory Peptides*.

[16] Hadjiivanova C., Belcheva S., Belcheva I. (2003). Cholecystokinin and learning and memory processes. *Acta physiologica et pharmacologica Bulgarica*.

[17] Bengtsson P., Lundqvist G., Nilsson G. (1989). Inhibition of acid formation and stimulation of somatostatin release by cholecystokinin-related peptides in rabbit gastric glands. *The Journal of Physiology*.

[18] Tirassa P., Costa N., Aloe L. (2005). CCK-8 prevents the development of kindling and regulates the GABA and NPY expression in the hippocampus of pentylenetetrazole (PTZ)-treated adult rats. *Neuropharmacology*.

[19] Li F., Li S., Liu Y., Cao K., Yang M. (2016). Effect of Heweianshen decoction on orexin-A and cholecystokinin-8 expression in rat models of insomnia. *Evidence-Based Complementary and Alternative Medicine*.

[20] Adamantidis A. R., Zhang F., Aravanis A. M., Deisseroth K., de Lecea L. (2007). Neural substrates of awakening probed with optogenetic control of hypocretin neurons. *Nature*.

[21] Ferrari L. L., Park D., Zhu L., Palmer M. R., Broadhurst R. Y., Arrigoni E. (2018). Regulation of lateral Hypothalamic orexin activity by local GABAergic neurons. *The Journal of Neuroscience*.

[22] Krowicki Z. K., Burmeister M. A., Berthoud H.-R., Scullion R. T., Fuchs K., Hornby P. J. (2002). Orexins in rat dorsal motor nucleus of the vagus potently stimulate gastric motor function. *American Journal of Physiology-Gastrointestinal and Liver Physiology*.

[23] Zhou Q. H., Zhou X. L., Xu M. B. (2018). Suanzaoren formulae for insomnia: updated clinical evidence and possible mechanisms. *Frontiers in Pharmacology*.

[24] Yi P.-L., Tsai C.-H., Chen Y.-C., Chang F.-C. (2007). Gamma-aminobutyric acid (GABA) receptor mediates suanzaorentang, a traditional Chinese herb remedy, -induced sleep alteration. *Journal of Biomedical Science*.

[25] Zhao Y., Liu Y., Lan X. M. (2016). Effect of *Dendrobium officinale* extraction on gastric carcinogenesis in rats. *Evidence-Based Complementary and Alternative Medicine*.

[26] Zhang Y., Wang H., Mei N. (2018). Protective effects of polysaccharide from *Dendrobium nobile* against ethanol-induced gastric damage in rats. *International Journal of Biological Macromolecules*.

[27] Zhu L., Wang Z., Zhai X. (2017). Simultaneous quantitative determination of 13 active components in the traditional Chinese medicinal preparation suanzaoren oral liquid by HPLC coupled with diode array detection and evaporative light scattering detection. *Journal of Separation Science*.

[28] Shi R., Han Y., Yan Y. (2019). Loganin exerts sedative and hypnotic effects via modulation of the serotonergic system and GABAergic neurons. *Frontiers in Pharmacology*.

[29] Fernandes J., Baliego L. G. Z., Peixinho-Pena L. F. (2013). Aerobic exercise attenuates inhibitory avoidance memory deficit induced by paradoxical sleep deprivation in rats. *Brain Research*.

[30] Han X.-Y., Wang Y.-N., Dou D.-Q. (2018). Regulatory effects of poria on substance and energy metabolism in cold-deficiency syndrome compared with heat-deficiency syndrome in rats. *Chinese Journal of Natural Medicines*.

[31] Yujra V. Q., Moreira Antunes H. K., Mônico-Neto M. (2020). Paradoxical sleep deprivation induces differential biological response in rat masticatory muscles: inflammation, autophagy and myogenesis. *Journal of Oral Rehabilitation*.

[32] Zhong Y., Zheng Q., Hu P. (2019). Sedative and hypnotic effects of compound anshen essential oil inhalation for insomnia. *BMC Complementary and Alternative Medicine*.

[33] Zieminska E., Toczylowska B., Diamandakis D. (2018). Glutamate, glutamine and GABA levels in rat brain measured using MRS, HPLC and NMR methods in study of two models of autism. *Frontiers in Molecular Neuroscience*.

[34] Lei S., Bo L., Chen Y. (2019). *Dendrobii officinalis*, a traditional Chinese edible and officinal plant, accelerates liver recovery by regulating the gut-liver axis in NAFLD mice. *Journal of Functional Foods*.

[35] Yang K., Lu T., Zhan L. (2020). Physicochemical characterization of polysaccharide from the leaf of *Dendrobium officinale* and effect on LPS induced damage in GES-1 cell. *International Journal of Biological Macromolecules*.

[36] Huangfu Y.-r., Peng W., Guo B.-j. (2019). Effects of acupuncture in treating insomnia due to spleen-stomach disharmony syndrome and its influence on intestinal microbiome: study protocol for a randomized controlled trial. *Journal of Integrative Medicine*.

[37] Liu Y., Lin C., Wu H., Wang X., Zhu Y. (2015). Acupuncture treatment of insomnia based on the spleen and stomach theory. *Journal of Integrative Medicine*.

[38] Zou Z., Han B., Gong M., Wang S., Liang S. (2014). NMR-based metabonomic studies on stomach heat and cold syndromes and intervention effects of the corresponding formulas. *Evidence-Based Complementary and Alternative Medicine*.

[39] Yannakoulia M., Anastasiou C. A., Karfopoulou E., Pehlivanidis A., Panagiotakos D. B., Vgontzas A. (2017). Sleep quality is associated with weight loss maintenance status: the medweight study. *Sleep Medicine*.

[40] Fernandez Hurst N., Bibolini M. J., Roth G. A. (2019). Diazepam inhibits proliferation of lymph node cells isolated from rats with experimental autoimmune encephalomyelitis. *Neuroimmunomodulation*.

[41] Li C., Mai Y., Dong M. (2019). Multivariate pattern classification of primary insomnia using three types of functional connectivity features. *Frontiers in Neurology*.

[42] Tranoulis A., Georgiou D., Soldatou A., Triantafyllidi V., Loutradis D., Michala L. (2019). Poor sleep and high anxiety levels in women with functional hypothalamic amenorrhoea: a wake-up call for physicians?. *European Journal of Obstetrics & Gynecology and Reproductive Biology: X*.

[43] Guo J. S., Chau J. F. L., Cho C. H., Koo M. W. L. (2005). Partial sleep deprivation compromises gastric mucosal integrity in rats. *Life Sciences*.

[44] Zuo J. X., Li M., Jiang L. (2020). Hydrogen sulfide prevents sleep deprivation-induced hippocampal damage by upregulation of sirt1 in the hippocampus. *Frontiers in Neuroscience*.

[45] Villarreal D. M., Derrick B., Vathy I. (2008). Prenatal morphine exposure attenuates the maintenance of late LTP in lateral perforant path projections to the dentate gyrus and the CA3 region in vivo. *Journal of Neurophysiology*.

[46] Kim S., Jo K., Hong K. B., Han S. H., Suh H. J. (2019). GABA and l-theanine mixture decreases sleep latency and improves NREM sleep. *Pharmaceutical Biology*.

[47] Jarry H., Hirsch B., Leonhardt S., Wuttke W. (1992). Amino acid neurotransmitter release in the preoptic area of rats during the positive feedback actions of estradiol on LH release. *Neuroendocrinology*.

[48] Toossi H., Del Cid-Pellitero E., Jones B. E. (2017). Homeostatic regulation through GABA and acetylcholine muscarinic receptors of motor trigeminal neurons following sleep deprivation. *Brain Structure and Function*.

[49] Volgin D. V., Lu J. W., Stettner G. M. (2014). Time- and behavioral state-dependent changes in posterior hypothalamic GABAA receptors contribute to the regulation of sleep. *PLoS One*.

[50] Gottesmann C. (2002). GABA mechanisms and sleep. *Neuroscience*.

[51] Abdou A. M., Higashiguchi S., Horie K., Kim M., Hatta H., Yokogoshi H. (2006). Relaxation and immunity enhancement effects of *γ*-aminobutyric acid (GABA) administration in humans. *Biofactors*.

[52] Shulkes A., Chick P., Hardy K. J. (1987). Nature of the gastric acid-gastrin feedback loop in the fetal sheep. *Clinical and Experimental Pharmacology and Physiology*.

[53] Caso J., Leza J., Menchen L. (2008). The effects of physical and psychological stress on the gastrointestinal tract: lessons from animal models. *Current Molecular Medicine*.

[54] Wang L., Song Y., Li F. (2014). Effects of Wen Dan Tang on insomnia-related anxiety and levels of the brain-gut peptide ghrelin. *Neural Regeneration Research*.

[55] Lanza M., Makovec F. (2000). Cholecystokinin (CCK) increases GABA release in the rat anterior nucleus accumbens via CCK B receptors located on glutamatergic interneurons. *Naunyn-Schmiedeberg’s Archives of Pharmacology*.

[56] Takahashi N., Okumura T., Yamada H., Kohgo Y. (1999). Stimulation of gastric acid secretion by centrally administered orexin-A in conscious rats. *Biochemical and Biophysical Research Communications*.

[57] Ford G. K., Al-Barazanji K. A., Wilson S., Jones D. N. C., Harbuz M. S., Jessop D. S. (2005). Orexin expression and function: glucocorticoid manipulation, stress, and feeding studies. *Endocrinology*.

