# Supplementary material for: Soporific Effect of Modified Suanzaoren Decoction and Its Effects on the Expression of CCK-8 and Orexin-A
Source: Evid Based Complement Alternat Med. 2020 Jun 16;2020:6984087. doi: 10.1155/2020/6984087 (PMC7315314; doi:10.1155/2020/6984087)
Supplement: Supplementary Materials — This experiment is mainly divided into the following three parts: first, using HPLC to detect the main ingredients in modified Suanzaoren decoction; second, evaluating its hypnotic effect through the pentobarbital-induced animal model of insomnia; and finally, establishing insomnia related to gastrointestinal diseases through sleep deprivation for 4 days and administration of hot drugs for 7 days, to evaluate the hypnotic effect of the modified Suanzaoren decoction and its possible mechanism. [file 6984087.f1.pdf]

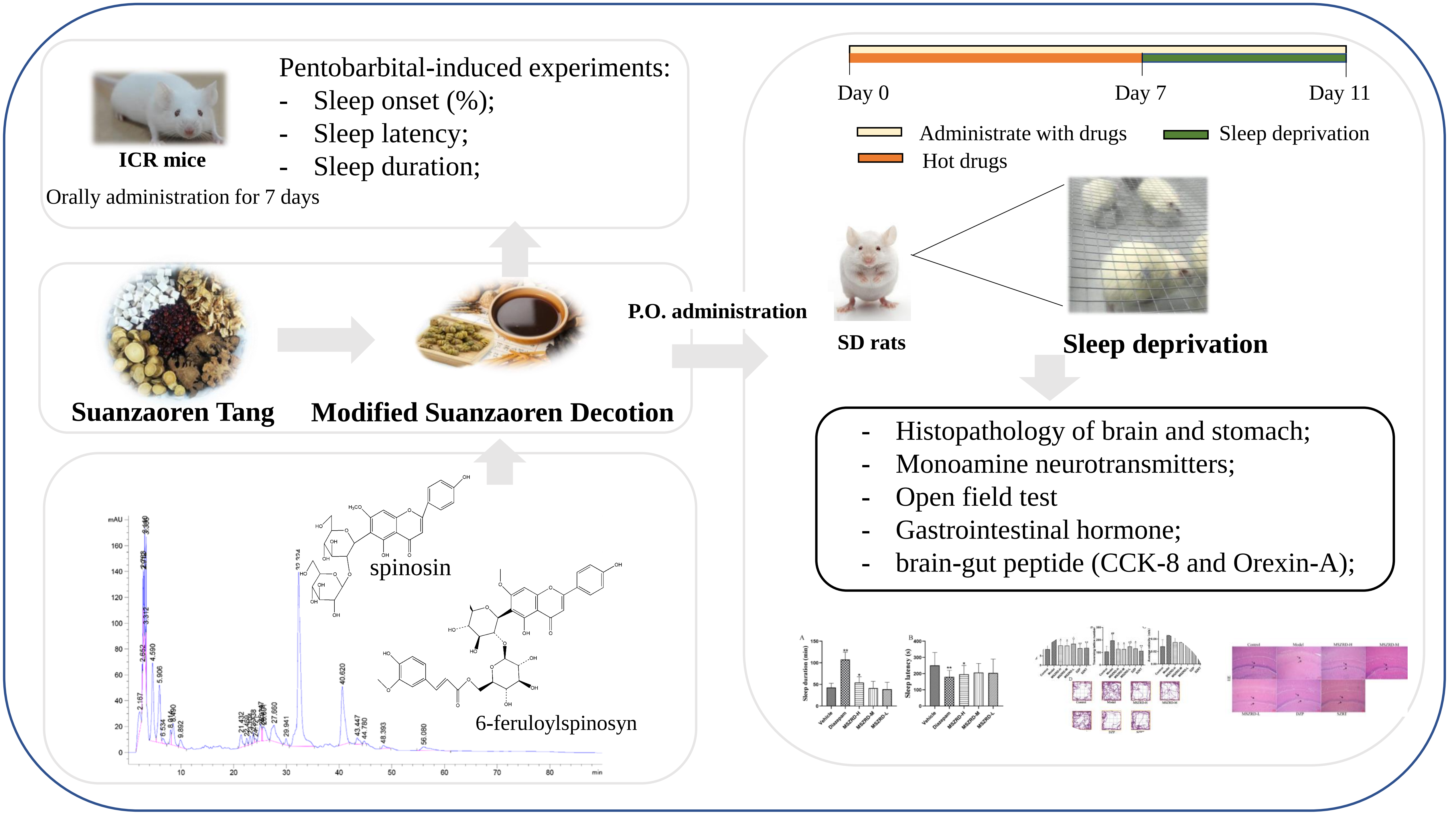

Pentobarbital-induced experiments:
- Sleep onset (%);
- Sleep latency;
- Sleep duration;
ICR mice
Orally administration for 7 days
Suanzaoren Tang
Modified Suanzaoren Decotion
P.O. administration
spinosin
6-feruloylspinosyn
Day 0
Day 7
Day 11
Administrate with drugs
Sleep deprivation
Hot drugs
SD rats
Sleep deprivation
- Histopathology of brain and stomach;
- Monoamine neurotransmitters;
- Open field test
- Gastrointestinal hormone;
- brain-gut peptide (CCK-8 and Orexin-A);
